# Herpes simplex virus, *APOE* ε4, and olfactory function in older adults: A cohort study

**DOI:** 10.1177/25424823261470737

**Published:** 2026-07-27

**Authors:** Eva-Stina Hemmingsson, Jan Olsson, Maria Josefsson, Fredrik Elgh, Maria Larsson, Bodil Weidung, Hugo Lövheim

**Affiliations:** 1 Department of Community Medicine and Rehabilitation, Geriatric Medicine, 249158 Umeå University, Umeå, Sweden; 2 Department of Clinical Microbiology, 8075 Umeå University, Umeå, Sweden; 3 Department of Statistics, USBE, 8075 Umeå University, Umeå, Sweden; 4 Centre for Demographic and Ageing Research, Umeå University, Umeå, Sweden; 5 Umeå Centre for Functional Brain Imaging (UFBI), Umeå University, Umeå, Sweden; 6 Gösta Ekman Laboratories, Department of Psychology, 162092 Stockholm University, Stockholm, Sweden; 7 Department of Public Health and Caring Sciences, Clinical Geriatrics, 174463 Uppsala University, Uppsala, Sweden

**Keywords:** Alzheimer's disease, *APOE* ε4, cohort study, herpes simplex virus, major neurocognitive disorder, olfaction

## Abstract

**Background:**

Olfactory dysfunction (OD) is associated with neurodegenerative disorders, including Alzheimer's disease (AD). Herpes simplex virus type 1 (HSV1) may increase AD risk, but longitudinal changes in olfaction related to HSV1 carriage and apolipoprotein ε4 (*APOE* ε4) status remain unclear.

**Objective:**

To investigate if OD is associated with carriage of HSV1, and whether *APOE* ε4 status modulates this possible association.

**Methods:**

From a large population-based cohort study in northern Sweden (Betula study), 1288 individuals with a baseline odor identification assessment were included, with follow-up assessment (n = 950) after five or ten years. Odor sensitivity (odor threshold test) was tested at a single time point for 651 individuals. HSV1 carriership and apolipoprotein E genotype were established for all participants.

**Results:**

Weighted linear mixed-model analyses showed that carriage of HSV1, regardless of *APOE* ε4 status, was not associated with decline in odor identification. At baseline, male gender and higher age were associated with impaired odor identification, while better semantic memory was associated with better odor identification ability. Higher age, low education level and better semantic memory were associated with poorer odor identification performance over time. Furthermore, multiple linear regression showed that male gender, higher age and low education level were significantly associated with impaired odor sensitivity.

**Conclusions:**

HSV1 did not associate with OD in this population-based cohort study with 5–10 years of follow-up, where olfaction was measured and analyzed using two different objective measurements. The association between OD and neurodegenerative diseases needs further investigation.

## Introduction

Olfactory dysfunction (OD) is common in the older population.^1^ The impairment often has a negative impact on the quality of life and is related to affective disorders such as anxiety and depression.^2^ OD is also associated with neurocognitive disorders including Parkinson's disease and Alzheimer's disease (AD).^3,4^ Approximately 85% of individuals with mild AD exhibit OD^5^ and research shows that OD constitutes a preclinical marker for impending cognitive decline, conversion to dementia, and increased mortality risk.^6–10^ In persons with AD, odor identification is typically more affected than measurements of odor sensitivity (i.e., odor thresholds, the lowest concentration at which a person can detect a scent).^4^ One explanation for this difference is that odor identification poses higher cognitive demands.^11^ The etiology of OD is heterogeneous, and risk factors include higher age, male sex, genetic background, nasal polyps, cardiovascular disease, diabetes, and exposures to viral infections.^12–14^

During the past decade, research has indicated that virus infections play a role in the development of AD, and the ubiquitous herpes simplex virus type 1 (HSV1) has been particularly highlighted.^15^ Epidemiological studies have shown that both carriership of the HSV and reactivation of HSV infections are associated with incident AD^16–19^ and dementia.^20^ Interestingly, an interaction has been found between HSV infection and genetic factors, with the apolipoprotein E allele 4 gene (*APOE* ε4) in combination with HSV increasing the risk of AD.^21–23^

Carriage of *APOE* ε4, which is strongly linked to AD, has also been associated with OD in cognitively healthy elderly adults.^24–27^ The ApoE-protein expressed in the olfactory epithelium and olfactory bulb is suggested to facilitates olfactory nerve regeneration.^28,29^ A dysfunctional ApoE-protein could delay repair and could possibly explain OD seen in different neurological disorders.^28^ Individuals diagnosed with AD with one ε4 allele experience odor identification decline, and even greater impairment is seen in homozygotic ε4 carriers.^30^ In postmortem studies of persons with AD, amyloid-β (Aβ) has been found in the olfactory epithelium^31^ and brain areas crucial to olfactory processing show early AD pathology,^32,33^ suggesting that impaired olfaction could reflect the disease progress of AD.^34^ Prior findings also support that *APOE* genotype and olfactory performance have a combined effect on dementia risk.^35,36^

Since OD may signal the onset of AD and HSV1 might contribute to the development of AD, it is of interest to explore whether there is a connection between HSV infection and olfactory function. Case reports also suggest that the olfactory pathway might play a role in the spread of herpetic meningoencephalitis.^37^ The relationship between HSV1 and olfactory function, particularly its interaction with *APOE* ε4, has not been previously explored. Thus, in this large population-based cohort study, we aimed to examine both single point assessment and longitudinal associations between HSV1 exposure and olfactory function measured through odor identification and odor threshold tests. Furthermore, we aimed to investigate whether *APOE* ε4 status modulated these possible associations.

## Methods

### Participants

Participants considered for inclusion in the present study were participants in the Betula study,^38–40^ recruited from the municipality of Umeå located in Northern Sweden, at the first and second wave of data collection (T1: 1988–1990 and T2: 1993–1995). The Betula study is a large population-based cohort initiated in 1988 at Umeå University, Sweden. At recruitment, the samples comprised 1000 individuals each, evenly distributed over 10 age cohorts ranging from 35–85 years of age. At recruitment at T1, 1976 persons were contacted to obtain 1000 individuals. The overall aim of the Betula study was to study how memory and health develop during adulthood.^38–40^ Participants were assessed every five years, and they underwent comprehensive examinations including a broad battery of cognitive tests, provided blood samples, and were interviewed about health, social, and economic status. Selection and testing procedures are described in detail in earlier studies.^16,40^ Participants gave informed consent, and the study was approved by the Regional Ethical Review Board in Umeå, Sweden (2017/17–31).

### Odor identification and detection sensitivity

Olfaction was first evaluated at the third wave of data collection of the Betula study (T3: 1998–2000), and later on at wave four (T4: 2003–2005) and wave five (T5: 2008–2010) of data collection. Therefore, for the present analyses, the first time a participant completed a modified version of the Scandinavian Odor Identification Test (SOIT)^41^ assessment (most often at T3) served as the baseline even though individuals were initially included earlier (at T1 or T2) in the Betula study. SOIT consists of 13 different odor stimuli that are familiar to the Scandinavian population (i.e., cinnamon, orange, vanilla, apple, almond). The odors are fairly strong in intensity and represent a wide range of qualities (such as sweet, spicy, woody, citrus, and floral). For each of the presented odors, subjects are provided with a written list of four response alternatives from which they select the best match. Odor identification ability was scored by the number of correctly identified odors (range 0–13), with a higher score representing a better ability.

In the fifth wave of data collection of the Betula study (T5: 2008–2010), an odor threshold test was also included to assess odor sensitivity. Briefly, the test required participants to identify odorous sticks with 16 different n-butanol concentrations ranging from low to high. The threshold scores varied from 1 (low olfactory sensitivity) to 16 (high olfactory sensitivity). Two pairs of sticks were used at each concentration level: one stick with n-butanol and one containing only water (blank). The participant was instructed to decide which of the two stimuli “smelled stronger.” The presentation started at an intermediate level (8), and each stick was presented approximately 2 cm under the participant's nose.^42^ The order of the presentation of the blank and odorant was random. If the participant was able to discriminate the odorant from the blank, the same concentration level was presented again until the criterion of four correct responses in a row was met. If the participant failed, a stronger concentration was presented until the criterion was met.

### Serum analyses, herpes simplex virus, and apolipoprotein E ε4

Serum samples were collected and stored frozen in minus 80°C. Samples were thawed and serum anti-HSV immunoglobulin G (IgG) positivity was assessed using an in-house enzyme-linked immunosorbent assay, modified from that described by Juto and Settergren (1988).^43^ Anti-HSV IgG positive samples were subtyped to determine carriage of anti-HSV1 and anti-HSV2 using commercial enzyme-linked immunosorbent assay (HerpeSelect 1 and HerpeSelect2; FOCUS Diagnostics). The procedures and tests have been previously described in detail.^16,18^

*APOE* genotype was determined with a polymerase chain reaction-based assay previously described.^44^ For samples in which DNA was not available, an *APOE* ε4 phenotyping was performed, based on the plasma protein isoforms, using a specific enzyme-linked immunosorbent assay (Cat # K4699-100) (BioVision Inc., CA, USA).

### Covariates

Covariates were included based on characteristics previously shown in the literature to be associated with both the exposure (HSV) and OD outcomes: age, sex and educational level.

Odor identification draws on semantic memory, as it put demands on an individual's knowledge of a specific odor's name.^45^ To control for the semantic memory component, a test of vocabulary, Synonym Reasoning Blocks (SRB1),^46^ was included as a covariate. In this test, participants were instructed to match 30 low-frequency words to one of five alternatives (synonyms). The score was the number of correctly identified synonyms within seven minutes (range 0–30).

Participants were recruited in the Betula study during the first wave of Betula data collection (T1: 1988–1990; n = 632) or during the second test wave (T2: 1993–1995; n = 656). Thus, participants from T1 have been exposed to testing on one more occasion than those from T2. To control for potential effects related to previous testing experiences, “sample year” was included as a covariate in the statistical analyses.

Smoking was evaluated as a possible confounder.^47^ However, as there was no significant difference between current smokers and non-smokers regarding the different olfactory measures in the univariate models, smoking was excluded as a covariate in the final model.

### Study sample and participants’ characteristics

Inclusion criteria for the present study were participants: 1) recruited during T1 and T2 from the longitudinally followed cohorts in the Betula study, 2) who had been assessed for HSV1 and *APOE4* carriage, and 3) who completed at least one odor identification assessment in the third, fourth or fifth wave of data collection. A total of 1288 participants were included in the main analyses, 950 thereof contributed to longitudinal analysis (Figure 1). Of the 1288 participants, single time point data concerning odor detection threshold were obtained from 651 participants from wave five of data collection.

**Figure 1.**
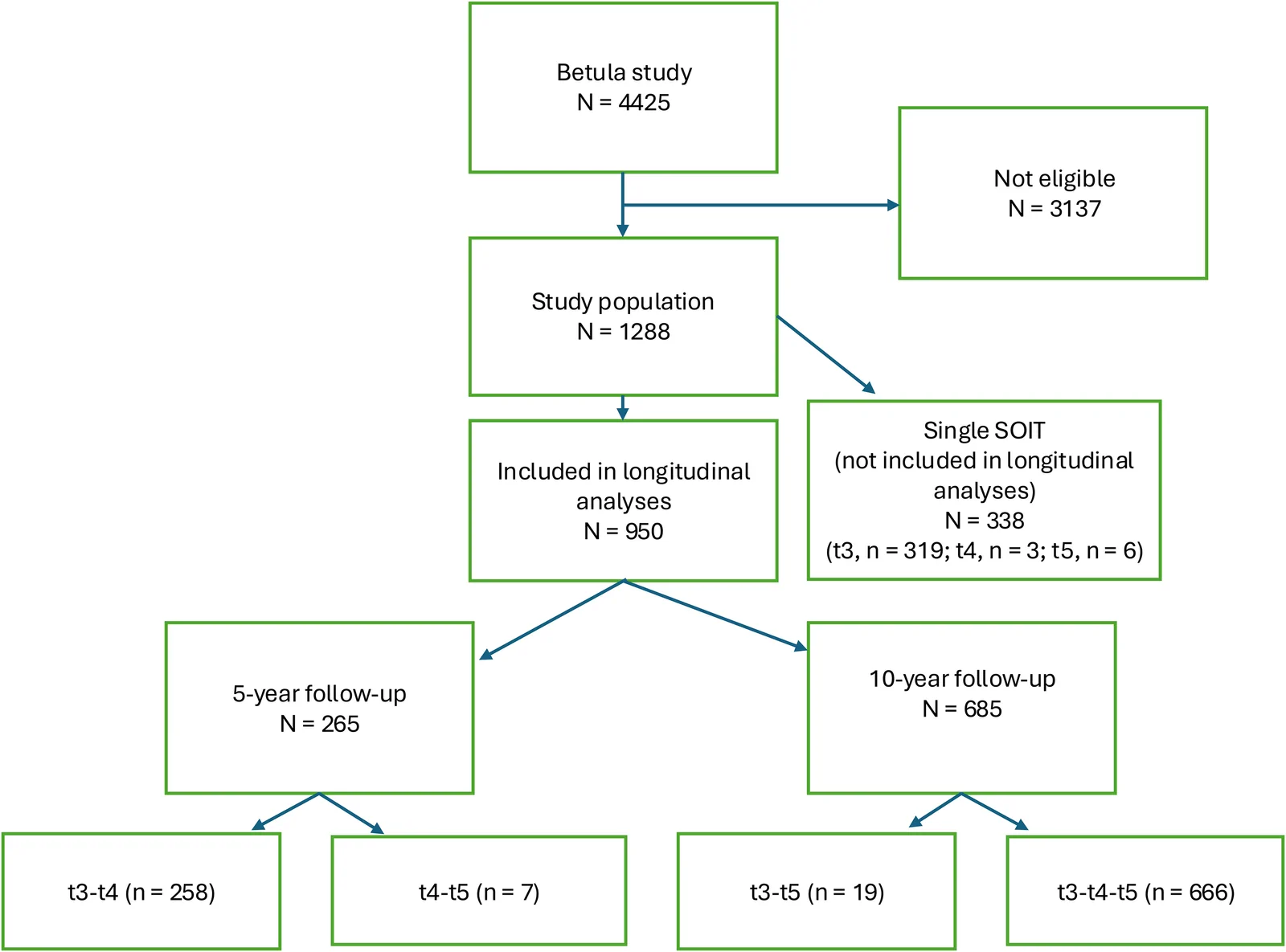
Flow chart of participants recruitment and follow-up.

### Statistical analysis

Student's T-test and Pearson's chi-square test were used to compare demographic measures among the two HSV1 IgG groups (carriers versus non-carriers). Odor identification, first assessed at the third wave (baseline), and across the five- to ten-year follow-up was analyzed as a continuous variable using weighted linear mixed models. Change in odor identification performance was compared between carriers of HSV1 and non-carriers, in combination with carriage of the *APOE* ε4 allele. Time in study, measured in years from baseline, was considered as a continuous covariate. Other covariates in the model were age, sex, years of education (stratified as low (6–7 years), medium (8–12) or high (>12 years) level), sample year, SRB1, carrier of anti-HSV1 IgG, carrier of *APOE* ε4, and interaction between HSV1 carriage and *APOE* ε4 carriage. Covariates were included as main effects and as interaction with time. To adjust for possible selection bias due to attrition before the third wave of data collection (when the odor assessment was first introduced), inverse probability weights were constructed. The probability of participation at wave 3 was estimated using baseline covariates, and weights were defined as the inverse of this probability. These weights were applied in both the cross-sectional linear regression models and the longitudinal mixed-effects models. Residual weights were included to compensate for any biases due to dropouts, estimated as the inverse probability of inclusion using linear regression and the full set of baseline variables. We specified a linear mixed-effects model including a subject-specific random intercept to account for within-person correlation across repeated measurements. No random slopes were included due to the limited number of repeated measurements (three time points).

Odor threshold was analyzed as a continuous variable using weighted multivariable linear regression. The regression model adjusted for age, sex, education, sample, SRB1, HSV1 IgG carriage, *APOE* ε4 carriage, and their interaction. To account for potential selection due to attrition prior to the odor threshold assessment at the fifth wave, inverse-probability weights were incorporated.

Statistical analyses were performed in R version 4.2.1. *p* ≤ 0.05 was considered to reflect significance.

## Results

Of the 1288 participants, 1082 (84.0%) were anti-HSV1 IgG antibody carriers. HSV1 carriers were more often females, were significantly older, and had lower levels of education. Among the participants, 309/1288 = 28.6% were *APOE* ε4 carriers, with no significant difference between HSV1 positive and HSV1 negative participants. Table 1 provides information on baseline characteristics of the study population.

**Table 1.** Sociodemographic characteristics of participants at baseline stratified by HSV1 status.

	Total	Anti-HSV1 IgG positive	Anti-HSV1 IgG negative
n (%)	1288	1082 (84.0)	206 (16.0)
Sex, female, n (%)	717 (55.7)	610 (56.4)	107 (51.9)
Age (y), mean ± SD	65.6 ± 12.3	66.7 ± 12.2	59.7 ± 11.2
Education (y), mean ± SD	10.1 ± 4.0	9.7 ± 3.8	11.9 ± 4.2
Current smoker, at T3, n (%)	81 (6.3)	64 (5.9)	17 (8.3)
Former smoker, at T3, n (%)	450 (34.9)	374 (34.7)	76 (36.9)
Odor identification (SOIT), mean ± SD	6.8 ± 2.3	6.8 ± 2.3	7.3 ± 2.2
Odor threshold, mean ± SD	5.2 ± 2.8	5.2 ± 2.9	5.3 ± 2.3
*APOE* ε4 carrier, n (%)	373 (29.0)	309 (28.6)	64 (31.1)

HSV1: herpes simplex virus type 1; IgG: immunoglobulin G; SOIT: Scandinavian Odor Identification Test; *APOE* ε4: allele 4 of apolipoprotein E; T3: Timepoint 3: the third wave of investigations in the Betula study 1998 to 2000.

There was a significant negative correlation between odor identification at baseline and change in odor identification at follow-up (*p* = <0.001). There was a significant positive correlation between odor identification at baseline and odor threshold (*p* = <0.001), but not between change in odor identification and odor threshold (*p* = 0.563).

### Analysis of odor identification performance

Results of the weighted linear mixed models are given in Table 2. Male subjects (*p* < 0.001), those with higher age (*p* < 0.001), and subjects from the cohort started at T2 (*p* = 0.035) showed significantly poorer odor identification ability at baseline. Better performance on the vocabulary test (SRB1) was related to significantly better odor identification ability at baseline (*p* < 0.001).

**Table 2.** Mixed-model analysis of HSV1 and odor identification, including the interaction between HSV1 and *APOE* ε4*.*

	Odor identification (SOIT)			
	Cross-sectional (covariates at intercept) N = 1288		Longitudinal (covariates × time) N = 950	
	Est (SE)	*p*	Est (SE)	*p*
Intercept	7.65 (0.26)	<0.001		
Time			−1.36 (0.38)	<0.001
HSV1 IgG carrier	0.21 (0.19)	0.27	−0.20 (0.27)	0.45
*APOE* ε4 carrier	0.31 (0.30)	0.30	−0.39 (0.42)	0.36
HSV1 IgG carrier × *APOE* ε4 carrier	−0.61 (0.33)	0.06	0.52 (0.47)	0.27
Male	−0.58 (0.11)	<0.001	0.13 (0.17)	0.44
Age (years)	−0.06 (0.01)	<0.001	−0.02 (0.01)	0.02
Cohort started at T2 versus T1	−0.23 (0.11)	0.04	0.35 (0.17)	0.04
Education low	0.12 (0.15)	0.41	−0.52 (0.24)	0.03
Education high	0.15 (0.15)	0.31	−0.12 (0.22)	0.58
SRB1	0.08 (0.01)	<0.001	−0.04 (0.02)	0.045

Reference is mean age, not *APOE* ε4 carrier, not HSV1 IgG carrier, woman, middle-level education, from cohort started at T1, and mean result at SRB at T3. SOIT: Scandinavian Odor Identification Test; HSV1: herpes simplex virus 1; IgG: immunoglobulin G; *APOE* ε4: allele 4 of apolipoprotein E; SRB1: synonym reasoning blocks.

Because the model included an interaction term, the main coefficient for HSV1 IgG represents the association among *APOE* ε4 non-carriers (the reference group). Among participants without *APOE* ε4, HSV1 IgG seropositivity was not associated with odor identification (β = 0.21, SE = 0.19, *p* = 0.27). The interaction between HSV1 IgG and *APOE-e4* showed a trend towards statistical significance (β = −0.61, SE = 0.33, *p* = 0.06) (Table 2), and among *APOE* ε4 carriers, the estimated association was negative (β = 0.21 − 0.61 = −0.40, SE = 0.27, *p* = 0.128), pointing in the direction of lower odor identification scores in HSV1-positive carriers.

HSV1 IgG seropositivity was not associated with odor identification change over time, neither among participants without *APOE* ε4 (β = −0.20, SE = 0.27, *p* = 0.45), nor among *APOE* ε4 carriers, (β = −0.20 + 0.52 = 0.32). The interaction between HSV1 IgG and *APOE* ε4 was not significant (β = 0.52, SE = 0.47, *p* = 0.27) (Table 2). However, it was found that higher age (*p* = 0.020), low education level (*p* = 0.030), and higher SRB1 (*p* = 0.045) were significantly associated with poorer odor identification ability over time (Table 2).

### Odor threshold

Results from the multivariable linear analysis on odor thresholds are summarized in Table 3. Since the model includes an interaction term, the main coefficient for HSV1 IgG represents the association among *APOE* ε4 non-carriers (the reference group). Among participants without *APOE* ε4, HSV1 IgG seropositivity was not associated with odor sensitivity (β = 0.08, SE = 0.37, *p* = 0.82).

**Table 3.** Multivariable linear regression analysis of HSV1 and odor threshold including interaction between HSV1 and *APOE* ε4.

	Odor threshold N = 651	
	Beta (standard error)	*p*
HSV1 IgG carrier	0.08 (0.37)	0.82
*APOE* ε4 carrier	−0.45 (0.60)	0.45
HSV1 IgG carrier × *APOE* ε4 carrier	−0.02 (0.66)	0.98
Male	−0.69 (0.23)	<0.001
Age (years)	−0.05 (0.01)	<0.001
Cohort started at T2 versus T1	−0.05 (0.23)	0.83
Education low	−1.15 (0.31)	<0.001
Education high	−0.28 (0.3)	0.34
SRB1	0.01 (0.03)	0.78

Reference is mean age, not *APOE* ε4 carrier, not HSV1 IgG carrier, woman, middle-level education, from cohort started at T1, and mean result at SRB at T3. HSV: herpes simplex virus; IgG: immunoglobulin G; *APOE* ε4: allele 4 of apolipoprotein E; SRB: Synonym Reasoning Blocks.

Among *APOEe4* carriers, the estimated association for HSV1 was similar (β = 0.08 − 0.02 = 0.06, SE = 0.55, *p* = 0.905). The interaction between HSV1 IgG and *APOE* ε4 was near zero (β = −0.02, SE = 0.66, *p* = 0.98), indicating that the presence of *APOE* ε4 did not modify the relationship between HSV1 and olfactory sensitivity. However, older age (*p* < 0.001), lower education level (*p* < 0.001), and male gender (*p* < 0.001) were significantly associated with lower odor sensitivity (Table 3).

Both regression models (odor identification and odor threshold, as above) were tested separately among men and women, and among participants younger than or older than 70 years respectively, with similar results in all subgroups (data not shown).

## Discussion

We aimed to study the association between HSV1 and olfactory function, and whether *APOE* ε4 status modulated this possible association. No evidence was found that carriage of HSV1 or *APOE* ε4, either individually or in combination, altered any of the investigated objective measures of olfactory function in this large-sized cohort study, with up to ten years of follow-up. Our result that carrying the *APOE* ε4 allele may not affect odor function contradicts some previous findings.^14^ Previous research suggested that *APOE* ε4 was associated with odor identification performance among elderly participants^26^ and also declined more rapidly in carriers compared to non-carriers.^27^ Therefore, in our relatively healthy and comparatively young cohort, *APOE* ε4 status may not yet have influenced odor identification performance.

In line with previous studies,^13,24,48^ age was associated with deterioration in both odor identification^14^ and odor threshold.^27^ Female participants performed significantly better than male participants in odor identification and odor threshold, which aligns with previous observations that females generally have better olfactory function than males.^2,14,49^ A meta-analysis by Sorokowski et al. (2019) suggested that sex-differences in olfaction could not be explained by a single factor but should be seen rather as multifactorial, including cognitive abilities, environmental exposures and hormonal differences.^49^

The olfactory nerve could provide one possible entry route for neurotropic viruses such as HSV1 into the brain,^50–53^ and an established link between OD and HSV1 would therefore indicate a possible route of entry to the brain and entorhinal cortex as a step in the process leading to HSV-associated AD. However, HSV1 carriage adjusted for covariates did not show any significant effect on odor identification ability either at baseline or over time, nor was there a significant effect on odor threshold. Even though viral infection is a known risk factor for OD, and HSV is known to be associated with neural lesions throughout the olfactory system,^54^ the present data do not support the hypothesis that HSV1 either alone or in combination with *APOE* ε4 carriage is associated with decline in olfactory function.

When put into the context of the suggested association between HSV1 and AD development, one explanation could be that HSV1 more often spreads from the trigeminal ganglion and its innervation of the meninges than through the olfactory pathway to reach the temporal and frontal cortices,^52,55^ or that the pathology of AD does not start within the peripheral olfactory regions but rather in the central olfactory regions.^32^ Periodic reactivation of HSV1 in the peripheral nervous system has also been suggested to cause CNS inflammation and longtime damage leading to neurodegeneration.^55^ Even though in our study population a majority were HSV1 seropositive, the virus could stay in latency among many participants not causing any harm, and associated OD changes might be too subtle to measure among the rather young and healthy participants.^55^ Another explanation could be that the previously seen association between OD and AD could partly be driven by individuals with Lewy body pathology,^56^ who in many studies were not sufficiently identified and separated from a pure AD group. In one study comparing olfactory function in subjects with neuropathologically confirmed dementia with Lewy bodies and AD, it was suggested that AD is associated with OD, but not without significant Lewy body pathology.^57^

### Strengths and limitations

The principal strengths of this study include the data obtained from a large population-based cohort with a long follow-up of up to 10 years with repeated olfactory tests. Olfaction was targeted by two different objective measurements. The HSV1 antibody data and *APOE* ε4 carriage data were established with high accuracy. Risk factors for OD are numerous^58^ and the risk of residual confounding cannot be omitted. Selective dropout may have affected our results since healthier participants might be more likely to remain in study, which might lead to an under-estimation of the deterioration in olfactory function.

### Conclusion

In this large-scale population-based cohort study, with up to ten years of follow-up, HSV1 carriage, either alone or in combination with *APOE* ε4 carriage, did not associate with decline in any of the investigated measures of olfactory function. The underlying cause of OD associated with neurodegenerative diseases needs further investigation.

## Data Availability

The data supporting the findings of this study are available on reasonable request. The data are not publicly available due to GDPR, privacy or ethical restrictions.

## References

[1] Doty RL Kamath V . The influences of age on olfaction: a review. Front Psychol 2014; 5: 20. 10.3389/fpsyg.2014.00020 PMC3916729 24570664

[2] Dan X Wechter N Gray S , et al. Olfactory dysfunction in aging and neurodegenerative diseases. Ageing Res Rev 2021; 70: 101416. 10.1016/j.arr.2021.101416 PMC8373788 34325072

[3] Mesholam RI Moberg PJ Mahr RN , et al. Olfaction in neurodegenerative disease: a meta-analysis of olfactory functioning in Alzheimer’s and Parkinson’s diseases. Arch Neurol 1998; 55: 84–90. 10.1001/archneur.55.1.84 9443714

[4] Silva MME Mercer PBS Witt MCZ , et al. Olfactory dysfunction in Alzheimer’s disease systematic review and meta-analysis. Dement Neuropsychol 2018; 12: 123–132. 10.1590/1980-57642018dn12-020004 PMC6022986 29988355

[5] Zou YM Lu D Liu LP , et al. Olfactory dysfunction in Alzheimer’s disease. Neuropsychiatr Dis Treat 2016; 12: 869–875. 10.2147/NDT.S104886 PMC4841431 27143888

[6] Stanciu I Larsson M Nordin S , et al. Olfactory impairment and subjective olfactory complaints independently predict conversion to dementia: a longitudinal, population-based study. J Int Neuropsychol Soc 2014; 20: 209–217. 10.1017/S1355617713001409 24451436

[7] Devanand DP Lee S Manly J , et al. Olfactory deficits predict cognitive decline and Alzheimer dementia in an urban community. Neurology 2015; 84: 182–189. 10.1212/WNL.0000000000001132 PMC4336090 25471394

[8] Roberts RO Christianson TJ Kremers WK , et al. Association between olfactory dysfunction and amnestic mild cognitive impairment and Alzheimer disease dementia. JAMA Neurol 2016; 73: 93–101. 10.1001/jamaneurol.2015.2952 PMC4710557 26569387

[9] Oltra J Ekström I Larsson M , et al. Olfactory dysfunction increases progression to dementia in cognitively impaired older adults: a 12-year population-based study. Geroscience 2026; 48: 591–603. 10.1007/s11357-025-01705-7 PMC12972283 40437282

[10] Ekström I Avelar-Pereira B Ruane R , et al. Olfactory decline profiles and their association with dementia conversion in an older population with 12 years of follow-up. Alzheimers Dement 2024; 20: e092262.

[11] Dulay MF Gesteland RC Shear PK , et al. Assessment of the influence of cognition and cognitive processing speed on three tests of olfaction. J Clin Exp Neuropsychol 2008; 30: 327–337. 10.1080/13803390701415892 17852612

[12] Doty RL . Olfactory dysfunction in neurodegenerative diseases: is there a common pathological substrate? Lancet Neurol 2017; 16: 478–488. 10.1016/S1474-4422(17)30123-0 28504111

[13] Schubert CR Cruickshanks KJ Klein BE , et al. Olfactory impairment in older adults: five-year incidence and risk factors. Laryngoscope 2011; 121: 873–878. 10.1002/lary.21416 PMC3063862 21298645

[14] Ekström I Larsson M Rizzuto D , et al. Predictors of olfactory decline in aging: a longitudinal population-based study. J Gerontol A Biol Sci Med Sci 2020; 75: 2441–2449. 10.1093/gerona/glaa221 PMC7662159 32886741

[15] Itzhaki RF Lathe R Balin BJ , et al. Microbes and Alzheimer’s disease. J Alzheimers Dis 2016; 51: 979–984. 10.3233/JAD-160152 PMC5457904 26967229

[16] Lovheim H Gilthorpe J Adolfsson R , et al. Reactivated herpes simplex infection increases the risk of Alzheimer’s disease. Alzheimers Dement 2015; 11: 593–599. 10.1016/j.jalz.2014.04.522 25043910

[17] Letenneur L Peres K Fleury H , et al. Seropositivity to herpes simplex virus antibodies and risk of Alzheimer’s disease: a population-based cohort study. PLoS One 2008; 3: e3637. 10.1371/journal.pone.0003637 PMC2572852 18982063

[18] Lovheim H Gilthorpe J Johansson A , et al. Herpes simplex infection and the risk of Alzheimer’s disease: a nested case-control study. Alzheimers Dement 2015; 11: 587–592. 10.1016/j.jalz.2014.07.157 25304990

[19] Liu Y Johnston C Jarousse N , et al. Association between herpes simplex virus type 1 and the risk of Alzheimer’s disease: a retrospective case-control study. BMJ Open 2025; 15: e093946. 10.1136/bmjopen-2024-093946 PMC12104942 40393802

[20] Vestin E Boström G Olsson J , et al. Herpes simplex viral infection doubles the risk of dementia in a contemporary cohort of older adults: a prospective study. J Alzheimers Dis 2024; 97: 1841–1850. 10.3233/JAD-230718 PMC10894565 38306033

[21] Itzhaki R . Herpes simplex virus type 1, apolipoprotein E and Alzheimer’ disease. Herpes 2004; 11: 77a–82a. 15319093

[22] Itzhaki RF Lin WR Shang D , et al. Herpes simplex virus type 1 in brain and risk of Alzheimer’s disease. Lancet 1997; 349: 241–244. 10.1016/S0140-6736(96)10149-5 9014911

[23] Lopatko Lindman K Weidung B Olsson J , et al. A genetic signature including apolipoprotein Eepsilon4 potentiates the risk of herpes simplex-associated Alzheimer’s disease. Alzheimers Dement (N Y) 2019; 5: 697–704. 10.1016/j.trci.2019.09.014 PMC6944738 31921962

[24] Larsson M Hedner M Papenberg G , et al. Olfactory memory in the old and very old: relations to episodic and semantic memory and APOE genotype. Neurobiol Aging 2016; 38: 118–126. 10.1016/j.neurobiolaging.2015.11.012 26827650

[25] Josefsson M Larsson M Nordin S , et al. APOE-varepsilon4 effects on longitudinal decline in olfactory and non-olfactory cognitive abilities in middle-aged and old adults. Sci Rep 2017; 7: 1286. 10.1038/s41598-017-01508-7 PMC5430873 28455505

[26] Olofsson JK Nordin S Wiens S , et al. Odor identification impairment in carriers of ApoE-varepsilon4 is independent of clinical dementia. Neurobiol Aging 2010; 31: 567–577. 10.1016/j.neurobiolaging.2008.05.019 18619712

[27] GoodSmith MS Wroblewski KE Schumm LP , et al. Association of APOE ε4 status with long-term declines in odor sensitivity, odor identification, and cognition in older US adults. Neurology 2023; 101: e1341–e1350. 10.1212/WNL.0000000000207659 PMC10558172 37495381

[28] Nathan BP Nannapaneni S Gairhe S , et al. The distribution of apolipoprotein E in mouse olfactory epithelium. Brain Res 2007; 1137: 78–83. 10.1016/j.brainres.2006.12.036 PMC1868464 17239830

[29] Nathan BP Nisar R Short J , et al. Delayed olfactory nerve regeneration in ApoE-deficient mice. Brain Res 2005; 1041: 87–94. 10.1016/j.brainres.2005.02.011 15804503

[30] Oleson S Murphy C . Olfactory dysfunction in ApoE varepsilon4/4 homozygotes with Alzheimer’s disease. J Alzheimers Dis 2015; 46: 791–803. 10.3233/JAD-150089 25869786

[31] Arnold SE Lee EB Moberg PJ , et al. Olfactory epithelium amyloid-beta and paired helical filament-tau pathology in Alzheimer disease. Ann Neurol 2010; 67: 462–469. 10.1002/ana.21910 PMC2864948 20437581

[32] Braak H Braak E . Neuropathological stageing of Alzheimer-related changes. Acta Neuropathol 1991; 82: 239–259. 10.1007/BF00308809 1759558

[33] Hyman BT Arriagada PV Van Hoesen GW . Pathologic changes in the olfactory system in aging and Alzheimer’s disease. Ann N Y Acad Sci 1991; 640: 14–19. 10.1111/j.1749-6632.1991.tb00184.x 1776730

[34] Murphy C . Olfactory and other sensory impairments in Alzheimer disease. Nat Rev Neurol 2019; 15: 11–24. 10.1038/s41582-018-0097-5 30532084

[35] Shrestha S Zhu X Griswold ME , et al. Olfaction and plasma biomarkers of Alzheimer disease and neurodegeneration in the atherosclerosis risk in communities study. Neurology 2025; 104: e213706. 10.1212/WNL.0000000000213706 PMC12083871 40373252

[36] Laukka EJ Ekström I Larsson M , et al. Markers of olfactory dysfunction and progression to dementia: a 12-year population-based study. Alzheimers Dement 2023; 19: 3019–3027. 10.1002/alz.12932 36689643

[37] Landis BN Vodicka J Hummel T . Olfactory dysfunction following herpetic meningoencephalitis. J Neurol 2010; 257: 439–443. 10.1007/s00415-009-5344-7 19820982

[38] Nilsson L-G Backkman L Erngrund K , et al. The betula prospective cohort study: memory, health, and aging. Aging Neuropsychol Cogn 1997; 4: 1–32.

[39] Nilsson L-G Adolfsson R Bockman L , et al. Betula: a prospective cohort study on memory, health and aging. Aging Neuropsychol Cogn 2004; 11: 134–148.

[40] Nyberg L Boraxbekk CJ Sörman DE , et al. Biological and environmental predictors of heterogeneity in neurocognitive ageing: evidence from Betula and other longitudinal studies. Ageing Res Rev 2020; 64: 101184. 10.1016/j.arr.2020.101184 32992046

[41] Nordin S Brämerson A Lidén E , et al. The scandinavian odor-identification test: development, reliability, validity and normative data. Acta Otolaryngol 1998; 118: 226–234. 10.1080/00016489850154946 9583791

[42] Hummel T Sekinger B Wolf SR , et al. ‘Sniffin’ sticks’: olfactory performance assessed by the combined testing of odor identification, odor discrimination and olfactory threshold. Chem Senses 1997; 22: 39–52. 10.1093/chemse/22.1.39 9056084

[43] Juto P Settergren B . Specific serum IgA, IgG and IgM antibody determination by a modified indirect ELISA-technique in primary and recurrent herpes simplex virus infection. J Virol Methods 1988; 20: 45–55. 10.1016/0166-0934(88)90039-0 2840451

[44] Sundström A Marklund P Nilsson LG , et al. APOE Influences on neuropsychological function after mild head injury: within-person comparisons. Neurology 2004; 62: 1963–1966. 10.1212/01.wnl.0000129268.83927.a8 15184597

[45] Larsson M . Semantic factors in episodic recognition of common odors in early and late adulthood: a review. Chem Senses 1997; 22: 623–633. 10.1093/chemse/22.6.623 9455609

[46] Durman I . SRB:1. Psykologiforlaget, 1960.

[47] Siegel JK Wroblewski KE McClintock MK , et al. Olfactory dysfunction persists after smoking cessation and signals increased cardiovascular risk. Int Forum Allergy Rhinol 2019; 9: 977–985. 10.1002/alr.22357 PMC6730657 31365791

[48] Schlosser RJ Desiato VM Storck KA , et al. A community-based study on the prevalence of olfactory dysfunction. Am J Rhinol Allergy 2020; 34: 661–670. 10.1177/1945892420922771 PMC7705196 32345032

[49] Sorokowski P Karwowski M Misiak M , et al. Sex differences in human olfaction: a meta-analysis. Front Psychol 2019; 10: 242. 10.3389/fpsyg.2019.00242 PMC6381007 30814965

[50] Dando SJ Mackay-Sim A Norton R , et al. Pathogens penetrating the central nervous system: infection pathways and the cellular and molecular mechanisms of invasion. Clin Microbiol Rev 2014; 27: 691–726. 10.1128/CMR.00118-13 PMC4187632 25278572

[51] Esiri MM Tomlinson AH . Herpes simplex encephalitis. Immunohistological demonstration of spread of virus via olfactory and trigeminal pathways after infection of facial skin in mice. J Neurol Sci 1984; 64: 213–217. 10.1016/0022-510x(84)90039-x 6379105

[52] Mori I Nishiyama Y Yokochi T , et al. Olfactory transmission of neurotropic viruses. J Neurovirol 2005; 11: 129–137. 10.1080/13550280590922793 16036791

[53] Bello-Morales R Andreu S López-Guerrero JA . The role of herpes simplex virus type 1 infection in demyelination of the central nervous system. Int J Mol Sci 2020; 21: 5026. 10.3390/ijms21145026 PMC7404202 32708697

[54] Lee JC Nallani R Cass L , et al. A systematic review of the neuropathologic findings of post-viral olfactory dysfunction: implications and novel insight for the COVID-19 pandemic. Am J Rhinol Allergy 2021; 35: 323–333. 10.1177/1945892420957853 PMC10404900 32915650

[55] Laval K Enquist LW . The potential role of herpes simplex virus type 1 and neuroinflammation in the pathogenesis of Alzheimer’s disease. Front Neurol 2021; 12: 658695. 10.3389/fneur.2021.658695 PMC8055853 33889129

[56] Palmqvist S Rossi M Hall S , et al. Cognitive effects of Lewy body pathology in clinically unimpaired individuals. Nat Med 2023; 29: 1971–1978. 10.1038/s41591-023-02450-0 PMC10427420 37464059

[57] Beach TG Adler CH Zhang N , et al. Severe hyposmia distinguishes neuropathologically confirmed dementia with Lewy bodies from Alzheimer’s disease dementia. PLoS One 2020; 15: e0231720. 10.1371/journal.pone.0231720 PMC7176090 32320406

[58] Nordin S Bramerson A . Complaints of olfactory disorders: epidemiology, assessment and clinical implications. Curr Opin Allergy Clin Immunol 2008; 8: 10–15. 10.1097/ACI.0b013e3282f3f473 18188011

